# Endoplasmic Reticulum Chaperones and Oxidoreductases: Critical Regulators of Tumor Cell Survival and Immunorecognition

**DOI:** 10.3389/fonc.2014.00291

**Published:** 2014-10-27

**Authors:** Tomás Gutiérrez, Thomas Simmen

**Affiliations:** ^1^Department of Cell Biology, Faculty of Medicine and Dentistry, University of Alberta, Edmonton, AB, Canada

**Keywords:** endoplasmic reticulum, localization, redox, Ca^2+^ signaling, cancer, immunity

## Abstract

Endoplasmic reticulum (ER) chaperones and oxidoreductases are abundant enzymes that mediate the production of fully folded secretory and transmembrane proteins. Resisting the Golgi and plasma membrane-directed “bulk flow,” ER chaperones and oxidoreductases enter retrograde trafficking whenever they are pulled outside of the ER by their substrates. Solid tumors are characterized by the increased production of reactive oxygen species (ROS), combined with reduced blood flow that leads to low oxygen supply and ER stress. Under these conditions, hypoxia and the unfolded protein response upregulate their target genes. When this occurs, ER oxidoreductases and chaperones become important regulators of tumor growth. However, under these conditions, these proteins not only promote the folding of proteins, but also alter the properties of the plasma membrane and hence modulate tumor immune recognition. For instance, high levels of calreticulin serve as an “eat-me” signal on the surface of tumor cells. Conversely, both intracellular and surface BiP/GRP78 promotes tumor growth. Other ER folding assistants able to modulate the properties of tumor tissue include protein disulfide isomerase (PDI), Ero1α and GRP94. Understanding the roles and mechanisms of ER chaperones in regulating tumor cell functions and immunorecognition will lead to important insight for the development of novel cancer therapies.

## Introduction

The endoplasmic reticulum (ER) is the location of oxidative protein folding, a mechanism that enzymatically manufactures fully folded secretory and transmembrane proteins. These two groups of proteins make up about 10 and 20% of a typical mammalian proteome, respectively (1). Ribosomally produced polypeptides for these two groups of proteins are first targeted to the ER membrane, where they interact with the Sec61 protein translocation channel (translocon) using their signal peptide (2). At this location, polypeptides undergo cytosolic folding that continues during translocation to the ER lumen (3, 4). Subsequently, the interaction with immunoglobulin binding protein (BiP/GRP78), a major ER-lumenal chaperone, initiates the production of secretory and transmembrane proteins (5, 6). If polypeptides are glycosylated they subsequently interact with the lectin chaperones calnexin and calreticulin (7), as well as oxidoreductases including protein disulfide isomerase (PDI) and related family members such as ERp57 (8). The oxidizing activity of these proteins is kept intact by oxygen- or hydrogen-peroxide consuming oxidoreductases such as Ero1α (9). Thus, ER chaperones and oxidoreductases cyclically interact with the ongoing flow of polypeptides emerging from the translocon. The flow of these proteins is massive. Using a vesicular stomatitis virus G protein (VSVG) fusion with green fluorescent protein (GFP), it has been estimated to amount to 7,000 molecules per second for this model protein alone (10, 11).

This observation raises the question as to how cells handle this quantity of export and how they ensure that exported proteins are segregated from resident ER chaperones and oxidoreductases. Early research using glycosylated short peptides had indicated that ER–Golgi trafficking occurs via non-specific “bulk flow” (12). However, this intuitive model may not be correct, since positive signals are not only needed for export from the ER (13, 14), but also for transit toward the Golgi complex (15). Moreover, secretory proteins are actually actively excluded from retrograde trafficking, which describes the trafficking route from the Golgi complex back to the ER (15). Conversely, most ER oxidoreductases and chaperones are equipped with a C-terminal KDEL motif that serves to interact with the KDEL receptor, a retrieval receptor that re-establishes ER localization for proteins with such a motif (16, 17). Therefore, given these efficient retention mechanisms, it makes little sense that tumor immunorecognition should be influenced by ER-restricted chaperones and oxidoreductases, when this mechanism is dictated by the properties of the cell surface. Nevertheless, in a cancer setting, proteins of this group can become localized to the plasma membrane or even secreted (18). When this occurs, ER oxidoreductases and chaperones become important regulators of tumor growth, but also of tumor immune recognition. For instance, the escape of calreticulin from the ER leads to the generation of an “eat-me” signal on the surface of tumor cells (19). Surface BiP/GRP78 is a target for antibody-based experimental therapies as well (20, 21). Understanding how these proteins target to the plasma membrane could therefore lead to important insight for the development of immune-based cancer therapies.

## ER Retrieval of Chaperones and Oxidoreductases

To ensure their residence to the ER and their availability for further work on newly synthesized polypeptides, chaperones and oxidoreductases are continually recycled back to the ER (22). Lumenal ER chaperones and oxidoreductases use the lysine-based C-terminal KDEL sequence for this purpose to interact with the KDEL receptor, a sorting receptor that cycles between the Golgi complex and the ER, first discovered by the Pelham lab in 1990 (16, 17). The KDEL receptor is part of a group of transmembrane proteins that all retrieve luminal ER proteins (23). These transmembrane receptor proteins typically use cytosolic, C-terminal lysine-based motifs (KKXX) to travel from the Golgi complex to the ER on a retrograde trafficking route (24). One example is the KKFF motif in the case of the lectin ERGIC53 (25).

KKXX motifs retrieve ER transmembrane proteins by mediating interaction with the coatomer protein complex, also called COPI (26). This is a multi-subunit protein complex composed of seven distinct proteins termed coatomer whose subunits are termed α, β, β′, γ, δ, ε, and ζ COPs (27). The binding of dilysine-bearing cargo molecules on α and β′ subunits nucleates the formation of COPI coats (28, 29). This step also requires the activation of ADP-ribosylation factor 1 (Arf1) (30). Upon pinching off from within the Golgi complex or the ER–Golgi intermediate compartment (ERGIC), retrograde vesicles are uncoated, following GTP hydrolysis on Arf1 mediated by Arf GTPase activating proteins (ArfGAPs) 1, 2, and 3 (31). These vesicles then migrate into the proximity of the ER. Here, a Soluble NSF Attachment Protein Receptor (SNARE) complex becomes important for retrograde trafficking. This trans-SNARE complex forms via Dsl1p (yeast) or Zw10 (mammals) with incoming COPI-derived vesicles (32, 33). These vesicles then fuse with the ER membrane at a site termed ER import sites, whose existence so far has only been demonstrated in plants (34). Trafficking from the Golgi complex to the ER is also under the control of Ras-related GTPases, members of a large regulatory protein family that serve as address tags for intracellular trafficking (35). Rab6 and Rab2 likely work in sequence to facilitate retrograde transport mediated by coatomer and directed to the ER (36–38), whereas Rab18 might regulate coatomer-independent trafficking from the Golgi to the ER (39). Together, the retention of ER chaperones and oxidoreductases clearly requires a large set of proteins, whose identity and mechanisms are now fairly well understood, despite some important outstanding questions (40).

In addition to COPI-mediated retrieval, some ER chaperones and oxidoreductases are retained in this organelle by other retention mechanisms (41). One type of mechanism requires the interaction of ER-resident proteins with COPI adaptors or helper proteins, exemplified by the interaction of a calnexin cytosolic acidic cluster motif with the sorting adaptor PACS-2 that dictates the extent of calnexin ER retention (42). This is particularly important, as calnexin does not have a canonical KKXX motif, but rather a di-arginine-based C-terminal motif involved in its retention (43).

Another way how ER transmembrane proteins are excluded from ER export is by the length of their transmembrane domains. This is demonstrated with artificial 17 transmembrane residue constructs that are unable to enter ER exit sites (ERES), whereas 22 residue long transmembrane domains allow for inclusion into Golgi-destined vesicles (44). The length of these transmembrane domains might facilitate inclusion into specific ER membrane domains (44). Some ER proteins use their transmembrane domains to enter a retrieval cycle similar to KDEL-tagged ER lumenal proteins. This is the case with sarcoendoplasmic reticulum calcium transport ATPase (SERCA) (45). Some of these proteins use the retrieval receptor Rer1 for their localization to the ER, as is the case for rhodopsin or components of the γ-secretase complex (46–48).

Endoplasmic reticulum lumenal chaperones and oxidoreductases further depend on the nature of the ER environment to achieve their typical distributions (49). This phenomenon is best understood for the ER oxidoreductase Ero1α (50). This lumenal ER protein lacks a KDEL motif, but uses interactions with other ER oxidoreductases (PDI and ERp44) to stay within the ER, but only under oxidizing conditions (51, 52). A similar mechanism is used by peroxiredoxin 4 (53). Less is known about the ability of Ca^2+^ binding domains to assist to ER retention, as is known to occur in the case of the ER chaperone calreticulin (54). While the depletion of ER lumenal Ca^2+^ is a known inducer of ER stress, the disruption of calreticulin ER localization is uniquely dependent on Ca^2+^. Calreticulin is not secreted upon induction of an ER stress with, for instance, tunicamycin (55). Potentially, this finding could indicate a requirement of Ca^2+^ binding to achieve a retrievable conformation of ER chaperones and oxidoreductases and specifically calreticulin. Such a hypothesis would be consistent with known alterations of protein conformation upon the loss of bound Ca^2+^ (56) and a general loss of chaperone–protein interactions within the ER upon the loss of free Ca^2+^ (57, 58). Either consequence could then lead to a loss of KDEL retrieval, either via masking of the KDEL sequence or via saturation of KDEL receptors (59). A similar Ca^2+^-dependent mechanism appears to determine the retention of BiP/GRP78 in the ER (60, 61). Together, ER localization of chaperones and oxidoreductases is lost or reduced upon the interference with retrieval receptors, upon modulation of the oxidative conditions of the ER and upon loss of ER Ca^2+^.

## ER Chaperones and Oxidoreductases on the Plasma Membrane of Tumor Cells

At first glance, the ER retention of chaperones and oxidoreductases appears like an abstract problem of interest only to very specialized cell biologists. Although cell types such as thyrocytes and immature thymocytes retain ER chaperones and oxidoreductases less efficiently, it is not known what the exact biological significance of this finding is (62, 63). However, over the past few years, information has emerged that ER chaperone and oxidoreductase retention in the ER is a critical sentinel mechanism that signals ER stress to the immune system (64). This is not unexpected, since ER chaperones such as calreticulin are functionally linked to the immune system and mediate the folding of major histocompatibility complex (MHC) class I (64). Through this function, ER chaperones and oxidoreductases already exhibit a tight link to the immune system via the regulation of intracellular peptide presentation by MHC class I on the plasma membrane (65). Accordingly, lost retention of ER chaperones and oxidoreductases upon ER stress impairs MHC class I expression on the surface (66). Surprisingly, however, this is not the only consequence. Calreticulin is normally enriched on the rough ER (rER) (67, 68). However, in cells undergoing ER stress, in particular following the depletion of ER Ca^2+^, calreticulin, PDI, BiP/GRP78, and GRP94 escape ER retention and retrieval (69). These cell surface-exposed chaperones and oxidoreductases can present antigens to the immune system (calreticulin, GRP94), serve as anchors for leukocytes (PDI), but can also activate pro-survival signaling pathways (BiP/GRP78) (18). In addition, ER stress generated from lost ER localization of chaperones and oxidoreductases leads to mitochondrial dysfunction and also triggers the activation of the NLRP3 inflammasome (70). In an organism, these metabolic and chemical changes lead to increased blood flow and leukocyte delivery to cells, where ER stress occurs (71). Therefore, the mechanisms that retain ER chaperones and oxidoreductases communicate ER stress to the immune system via multiple readouts: they determine MHC class I surface exposure, they influence the activation of inflammation, but they also signal the intracellular stress status to the immune system when found on the plasma membrane.

## Calreticulin: A DAMP on the Plasma Membrane of Tumor Cells

The appearance of ER chaperones and oxidoreductases on the plasma membrane corresponds to a danger-associated molecular pattern (DAMP) (72). DAMPs are molecules that are normally intracellular, but become exposed on the plasma membrane in stressed, damaged, or dying cells, as well as in tumor cells (73). Their presence on the cell surface leads to the recruitment of innate inflammatory cells, following the interaction of surface DAMPs with pattern-recognition receptors (PRRs) (74). An example for this is CD91, found on the surface of dendritic cells (DC) and other antigen-presenting cells (APC), which interacts with the ER-derived DAMPs calreticulin and GRP94 on stressed or dying cells (75). Upon formation of a complex between these proteins, a potent “eat-me” signal is generated and phagocytosis of calreticulin or GRP94-bearing stressed cells is initiated (19, 76). In contrast, CD47 acts as an inhibitor of this activity of calreticulin by interfering with the calreticulin–CD91 complex formation (64, 76).

**Figure 1:**
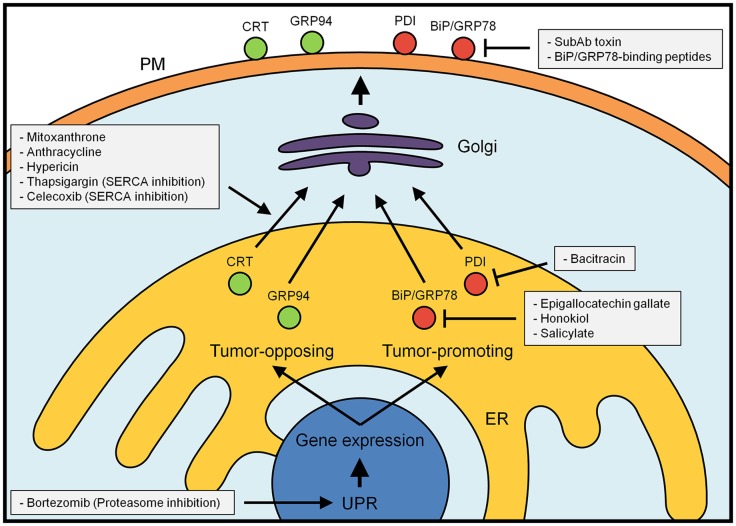
**Major tumor-promoting (red) and tumor-inhibitory (green) ER chaperones and oxidoreductases**. Their localization and function within the ER can be favorably influenced by a number of currently known drugs (for details see text). At the plasma membrane, inhibitory binding peptides can stop the tumor-promoting activity of this class of proteins. In contrast, simple modulation of the unfolded protein response (UPR) is expected to induce both tumor-promoting and tumor-blocking responses.

This mechanism is particularly important in the cancer scenario (Figure 1), where calreticulin is today one of the most extensively studied DAMPs that dictates the immunogenicity of cancer cells (19, 77). Importantly, calreticulin exposure on the plasma membrane is triggered upon treatment with different chemotherapeutic stimuli, including cisplatin and the anthracyclines doxorubicin, idarubicin, and mitoxantrone (19, 78). However, it is not clear whether calreticulin remains in the membrane of the stressed cell or is transferred over to immune cells (79). Regardless of the exact location of this extracellular calreticulin, stressed and apoptotic cells are subsequently engulfed and eliminated (80). This activation of the immune system can be exploited via the injection of calreticulin-coated cancer cells. Once in the blood stream, these abnormal cells can trigger a tumor-specific immune-response that eventually may activate an anti-tumor immune-response *in vivo* (81).

An additional important prerequisite for the immuno-elimination of tumor cells using the calreticulin “eat-me” signal is autophagy: the inhibition of autophagy significantly increases the amounts of calreticulin on the surface of stressed tumor cells, suggesting that autophagy-competent cancer tissue may be less susceptible to calreticulin-mediated immunorecognition of tumor cells (82, 83). In contrast, autophagy promotes the secretion of ATP upon ER stress, another tumor DAMP (84, 85). Interestingly, not only calreticulin on the plasma membrane, but also its overall expression is frequently enhanced in tumor tissue, potentially indicating that this chaperone could indeed provide an avenue for future cancer immunotherapy (86). Specifically, calreticulin over-expression is associated with the development and progression of pancreatic cancer (87). However, studies on infiltrating ductal breast carcinomas (IDCAs) were not able to detect an involvement of calreticulin in the development of a humoral immune-response (88). In defense of the calreticulin role as a protective mechanism against cancer, none of these studies have investigated the intracellular distribution of calreticulin in the respective tumor scenario. Consistent with this caveat, hepatocellular carcinoma has been found associated with high levels of circulating anti-calreticulin antibodies (89). In addition, serum IgG levels of anti-calreticulin autoantibodies have been found to be significantly higher in bladder cancer patients than in normal controls, leading to the proposal of anti-calreticulin antibodies as a novel biomarker for bladder cancer progression (90). It is currently unclear whether the injection of a fragment of recombinant calreticulin blocks tumor growth using these or other mechanisms (91, 92).

## ERp57, GRP94, Ero1α, and PDI: Functions Beyond Immunorecognition for Tumor Cell Migration

Other ER chaperones and oxidoreductases also show aberrant targeting to the plasma membrane. One example is ERp57, which is critical for the peptide loading complex for MHC class I together with calreticulin (93). Similar to what occurs with calreticulin, ERp57 also appears on the cell surface following anthracycline treatment. Importantly, ERp57 might not act as a DAMP itself, but rather as a prerequisite for calreticulin surface targeting (94, 95). The expression level of ERp57 in cancer does not provide much insight about its role in cancer, since bladder and gastric cancers appear to be characterized by low levels of calreticulin and ERp57 (96, 97).

GRP94 (also called gp96) is another prominent chaperone of the ER that has a much smaller set of client proteins when compared to calreticulin (98). Its substrates include toll-like receptors (TLRs), important sensors of DAMPs (99). This Hsp90 family protein can escape ER retention like calreticulin, and is found secreted from pancreatic cells and hepatocytes (100, 101). In contrast, tumor cells are decorated with surface-bound GRP94 (102, 103). On this localization, GRP94 acts as a DAMP similar to calreticulin (104) and in parallel to surface-exposed Hsp90 (105). In addition, GRP94 also binds HER2 on the surface of breast cancer cells, and regulates its cancer-promoting activity (106). Interestingly, cell surface GRP94 may interact with the CD91 receptor, like calreticulin, albeit with unclear functional significance (107, 108). Breast cancer tissue is characterized by the over-expression of GRP94 that may modulate the ability of tumor cells to migrate (109).

The oxidoreductase PDI is a central enzyme in the formation of disulfide bonds in secreted proteins (110). This protein also localizes in significant amounts to the cell surface of platelets, CHO, and pancreatic cells, as well as thyrocytes (62, 111–113). Here, it modulates surface-exposed thiols (113, 114) and cellular adhesion of immune cells via the association with integrins (115, 116). This mechanism also determines the ability of T helper cells to migrate through the extracellular matrix (117). PDI expression is tied to tumor vascularization that is often low and results in the activation of the hypoxia-dependent transcription factor HIF-1α (118). This transcription factor then promotes the upregulation of the oxidoreductases PDI and Ero1α (119–121). Subsequently, increased PDI and Ero1α expression also induces the production of vascular endothelial growth factor (VEGF), which, in turn, enables hypoxic tumors to improve angiogenesis (120, 121). Similar to GRP94, the levels of PDI and Ero1α have also been found to correlate with the invasiveness of glioma and the metastatic ability of soft tissue sarcoma, due to the role of PDI in mediating the interaction of cells with integrins (122, 123). Although Ero1α is secreted from hypoxic tumor cells, we currently do not known whether this occurs *in vivo* and what the function of surface or extracellular Ero1α is (67).

## BiP/GRP78, an Inhibitor of Tumor Cell Apoptosis and Immunorecognition

Compared to PDI, more is known about the role of BiP/GRP78 for cancer cells, and specifically when found on the plasma membrane. BiP/GRP78 is over-expressed in many cancers, a hallmark that is associated with aggressive growth, invasive properties, and therapeutic resistance (124). This chaperone is a major regulator of ER protein folding and ER stress (125). By binding hydrophobic surfaces on newly synthesized polypeptides, BiP/GRP78 is first in line for ER protein folding, a role that becomes accentuated when misfolded polypeptides accumulate within the ER. Under that condition, also termed ER stress, BiP/GRP78 binds to unfolded proteins in its ATP-bound form, mediates their folding at the expense of ATP and is released when GDP is exchanged with GTP (126, 127). Folding is typically achieved through multiple rounds of binding and release of BiP/GRP78. Interestingly, when BiP/GRP78 acts as a chaperone, it dissociates from the ER transmembrane stress sensor proteins inositol requiring enzyme 1 (Ire1), protein kinase RNA-like ER kinase (PERK), and activating transcription factor 6 (ATF6) that are then able to trigger the unfolded protein response (UPR) (128). This intracellular signaling pathway activates the transcription of numerous ER chaperones and oxidoreductases to protect the cell from accumulated unfolded proteins, but also acts as an activator of apoptosis (129). Notably, BiP/GRP78 itself is a transcriptional target of the UPR via ER stress-responsive elements that can bind to ATF6 (130).

In cancer tissue, the UPR is frequently constitutively active, because solid tumors are poorly vascularized, leading to low oxygen delivery for mitochondria and low glucose delivery for glycolysis, both a cause of low ATP availability for ER protein folding (131). Consistent with this, BiP/GRP78 has been found over-expressed in prostate, head and neck, melanoma, breast, lung, brain, gastric, colon, and hepatocellular carcinomas (132). High levels of BiP/GRP78 act first of all as suppressors of apoptosis (133, 134), based on its role as a suppressor of the UPR (18), but also from its ability to sequester ER-associated pro-apoptotic Bcl2 family proteins such as Bik (135). Over-expression of BiP/GRP78 also inhibits pro-apoptotic Ca^2+^ transfer from the ER to mitochondria in astrocytes. This likely occurs due to the inhibitory action of BiP/GRP78 on the inositol-1,4,5 trisphosphate receptors (IP3Rs), major Ca^2+^ release channels of the ER (136, 137). As expected from these tumor-promoting roles of BiP/GRP78, high levels of this ER chaperone lead to poor prognosis in breast cancer (138).

As a side effect, the UPR not only leads to elevated expression of BiP/GRP78 in tumor tissue, but also leads to aberrant localization of this ER chaperone to the cytosol, mitochondria, and the plasma membrane (124). Cell surface BiP/GRP78 is apparently directly tied to its expression level that is under the control of the UPR, suggesting that high expression of this chaperone leads to saturation of the KDEL receptor retrieval mechanism (61). This phenomenon has been found in prostate, ovarian, and gastric cancer, as well as melanoma (21, 139–141). In some tumors, so much BiP/GRP78 escapes from the ER that secretion results, accompanied by the production of autoantibodies (142). These autoantibodies can promote or inhibit proliferation and apoptosis, but also interfere with phosphoinositide 3-kinase (PI3K), Akt, and MAP kinase pathways with the consequence of increased survival in several types of tumors (143, 144). This latter activity depends on the activating, physical interaction of cell surface BiP/GRP78 with PI3K that subsequently results in the activation of its downstream target Akt (145, 146). This activity of surface BiP/GRP78 may depend on α2-macroglobulin (α2M*), since the association between the two proteins triggers Akt phosphorylation in a PI-3 kinase-dependent manner (147, 148). In contrast, low levels of BiP/GRP78 tend to have opposite effects in mice and result in decreased activity of PI3K signaling in prostate and leukemia cancer models (149, 150).

Like calreticulin, BiP/GRP78 also influences the way cancer cells interact with the immune system. However, whereas calreticulin provides an “eat-me” signal, cell surface BiP/GRP78 protects insulinoma and fibrosarcoma cells from cytotoxic T lymphocytes (151, 152). In addition, BiP/GRP78 also interacts with MHC class I on the cell surface, although the functional significance of this observation is currently unclear (153).

## Avenues of Interference with ER Chaperones in Cancer

Increased expression and cell surface appearance of ER chaperones and oxidoreductases have emerged as critical hallmarks of cancer cells and as consequences of low tumor vascularization that results in hypoxia. The observations outlined in our review suggest this insight may be used to develop new strategies to treat cancer (Figure 1) (154). In cancer, an approach under consideration consists in triggering the UPR (155). A number of compounds are currently in preclinical studies or Phase II/III trials and typically attempt to prevent the pro-survival readout of the UPR. This approach led to marked decrease of cancer growth in a multiple myeloma xenograft model (156). A promising strategy appears to be the combination of such drugs with bortezomib, a blocker of the proteasome and inducer of ER stress (157). With this combination of drugs, stress-inducing bortezomib primes cancer cells for death that becomes inevitable, once an inhibitor of protective UPR responses is added to the mix. A similar approach aims to target the redox-modulatory role of ER chaperones and oxidoreductases using the PDI inhibitor bacitracin, which acts as a potent booster of the chemotherapeutics fenretinide and bortezomib (158). Due to the toxicity of bacitracin, novel PDI inhibitors are currently under development (159). Another way how ER redox modulation can be used as an adjuvant for cancer chemotherapy is by interfering with the redox-sensitive activity of SERCA to allow for increased cell stress in tumor tissue due to reduced ER Ca^2+^ content (160, 161). Given the tumor-promoting (e.g., BiP/GRP78, PDI) and tumor-opposing (e.g., calreticulin) activities of ER chaperones and oxidoreductases, current knowledge suggests more pinpointed approaches are needed to increase efficacy of ER-targeted cancer chemotherapeutic strategies.

Interestingly, the SERCA inhibitor thapsigargin and the non-steroidal anti-inflammatory drug celecoxib both make use of the interference with ER Ca^2+^ content as a weapon against tumor cells (Figure 1) (162–164). Under this condition, calreticulin, PDI, BiP/GRP78, and GRP94 escape ER retention and retrieval (69, 165). This effect is similar to an indiscriminate activation of the UPR, which turns on ER chaperone and oxidoreductase production, and leads to the saturation of KDEL as well as di-lysine-based retrieval to the ER (166, 167). Similar to blanket interference with the UPR, this approach is bound to have tumor-promoting and tumor-opposing effects: while calreticulin and GRP94 will appear as immune system targets on the cell surface of tumor cells, PDI and BiP/GRP78 will have tumor-promoting effects as promoters of cell mobility and blockers of apoptosis.

Ideally, an efficient therapy would aim to generate cell surface calreticulin to serve as an efficient “eat-me” signal on tumor cells, while down-regulating or inactivating BiP/GRP78 on the plasma membrane, which acts as tumor-promoting. To achieve such a goal, it would be helpful to understand the molecular machinery of calreticulin plasma membrane exposure, currently known to require the triggering of PERK, the cleavage of caspase-8, and the functioning of SNARE proteins (168). Mitoxantrone, an anthracycline that robustly influences these mechanisms and leads to calreticulin surface exposure, is currently in clinical trials against lymphoma (Figure 1) (169).

In contrast to calreticulin, the requirements for BiP/GRP78 cell surface exposure are less understood. As a sole factor, the transmembrane protein MTJ-1 has been identified as critical for BiP/GRP78 surface translocation (170), possibly via the catalysis of ATP exchange through its J domain (171). Interestingly, photo-dynamic therapy (PDT) using the ER-localized photosensitizer hypericin may be such a magic bullet: not only does it result in the reduction of SERCA activity (172), but this treatment specifically results in the surface targeting of only calreticulin, and not BiP/GRP78 (84). Promisingly, this treatment causes tumor regression in BALB/c mice inoculated with colon carcinoma (173).

Conversely, inhibitory agents against BiP/GRP78 could create tumor cell specificity of UPR-targeted anti-cancer strategies (Figure 1). Approaches include the selective destruction of BiP/GRP78 on the surface with the bacterial toxin SubAb that cleaves and inactivates this chaperone (174). This strategy delayed the growth of multiple cancer xenografts in mice (175, 176). Cancer cells also respond well to the inhibition of the BiP/GRP78 ATPase activity with epigallocatechin gallate, honokiol, and salicylate (177–179). It is currently unknown whether these effects stem from inhibiting the activity of MTJ-1 that is needed to transport BiP/GRP78 to the plasma membrane (170). Xenograft growth of tumors is also inhibited in the presence of BiP/GRP78-binding peptides that obstruct the chaperone’s folding pocket (180). Importantly, these peptides bind specifically to tumor cells and abrogate their growth *in vivo* (181, 182). Such a strategy might be particularly important following the surgical removal of tumor tissue or in combination with chemotherapeutic approaches (183, 184).

## Conclusion

Endoplasmic reticulum chaperones and oxidoreductases have emerged as unlikely regulators of tumor growth. While neither being directly connected to the regulation of cell division and growth, nor the triggering of apoptosis, they instead frequently acquire new functions unrelated to their classic ER roles in a cancer setting. These new roles coincide with their relocation from the ER to the plasma membrane. In most cases, this occurs because the UPR triggers the production of more chaperones and oxidoreductases that eventually saturate the KDEL retrieval machinery. Once at the plasma membrane, ER chaperones and oxidoreductases serve as DAMPs for the immune system (calreticulin, GRP94) or modulators of tumor hallmarks (BiP/GRP78, PDI). The exploitation of this group of proteins as cancer therapeutic targets will require a detailed understanding of their intracellular and extracellular roles. Our current knowledge has identified chaperones that serve as DAMPs, whereas modulators of tumor hallmarks including cell death and metabolism are typically tumor-promoting. Specific triggers and inhibitors of the functions of ER chaperones and oxidoreductases will help direct cancer therapeutic approaches in the right direction. This insight warrants further investigation on this class of proteins.

## Conflict of Interest Statement

The authors declare that the research was conducted in the absence of any commercial or financial relationships that could be construed as a potential conflict of interest.
